# Efficacy of a school-based, universal prevention programme for depression and anxiety in adolescents (OurFutures Mental Health): a two-arm cluster-randomised controlled trial

**DOI:** 10.1016/j.eclinm.2025.103672

**Published:** 2025-11-28

**Authors:** Lucinda Grummitt, Siobhan O'Dean, Louise Birrell, Sasha Bailey, Isobel Ivison, Emily Hunter, An Nguyen, Erin Veronica Kelly, Lauren A. Gardner, Katrina E. Champion, Cath Chapman, Maree Teesson, Nicola C. Newton, Emma L. Barrett

**Affiliations:** a The Matilda Centre for Research in Mental Health and Substance Use, The University of Sydney, Sydney, NSW, Australia

**Keywords:** Mental health, Prevention, School, Randomised controlled trial

## Abstract

**Background:**

Adolescence is a critical period for the onset of mental health challenges. We evaluated the efficacy of a school-based intervention (*OurFutures Mental Health)* in promoting mental health knowledge and preventing symptoms of depression and anxiety.

**Methods:**

A two-arm cluster-randomised controlled trial was conducted from 2023 to 2024 with Year 8/9 students from 10 secondary schools in Australia. Schools were randomised (1:1) to the *OurFutures Mental Health* intervention or active control (usual health education) using the *Blockrand* function in R by an external statistician. Eligible participants were Year 8/9 students enrolled at participating schools. *OurFutures Mental Health* is a six-lesson, trauma-informed and gender- and sexuality-affirming intervention, employing a cognitive-behavioural approach. The three, pre-registered primary outcomes were mental health knowledge and depressive and anxiety symptoms at 3-months post-baseline. Linear mixed-effects regression models were run on the intention to treat sample, with random intercepts for participants and schools, tested whether receiving the intervention improved knowledge and reduced depression and anxiety symptoms relative to active control. The trial was registered with the Australian and New Zealand Clinical Trials Registry, ACTRN12622001582741.

**Findings:**

Between Dec 6, 2022 and April 12, 2024, we recruited 17 schools (1785 students), of which 10 schools (784 students) participated in the baseline assessment (mean age = 13.8 years; 467 [59.6%] male; intervention: 6 schools, 497 students; control: 4 schools, 287 students). Seven schools withdrew prior to baseline (6 control; 1 intervention). Knowledge scores were significantly higher in the intervention group post-intervention, but not at 3 months (β = 0.30, 95% CI: −0.32–0.91, p = 0.34). No significant intervention effects were observed for depressive symptoms at 3-months (β = −0.94, 95% CI: −1.88 to 0.04, p = 0.055). However, the intervention group showed a greater reduction in anxiety symptoms at 3-month follow-up compared to active control (β = −1.05, 95% CI: −1.93 to −0.12, p = 0.024). No adverse events were reported.

**Interpretation:**

*OurFutures Mental Health* may be an efficacious intervention to improve mental health knowledge and prevent anxiety symptoms among adolescents in the short-term. Limitations of this trial include high participant attrition and withdrawal of several schools post-randomisation, limiting generalisability. The trial was also underpowered, limiting detection of smaller intervention effects. Further research is needed to improve efficacy for prevention of depression.

**Funding:**

10.13039/501100016053Paul Ramsay Foundation.

**Research in context**

**Evidence before this study**

The evidence for universal, school-based interventions in preventing depression and anxiety among adolescents is mixed. We searched PubMed to identify studies assessing school-based prevention programmes for depression and anxiety published before July 16, 2022. Our search strategy included the terms: “adolescent or youth or teen” and “school” and “anxiety or depression or internalizing” and “prevention or intervention”. We considered studies that were either randomised controlled trials or systematic reviews/meta-analyses of these trials, focussing on universal interventions (delivered to all students) for adolescents aged 11–18 years, and that included a comparison group (no intervention, standard education, or an alternative programme). Meta-analytic results indicated universal depression and anxiety prevention approaches can lead to small, short-term reductions in depression and anxiety symptoms compared to control. However, findings from two recent, large-scale randomised controlled trials showed no significant effects of the intervention on depressive symptoms and some indication that the interventions had unintended iatrogenic effects.

**Added value of this study**

We conducted a cluster RCT to evaluate the short-term efficacy of the *OurFutures Mental Health* intervention in promoting mental health knowledge and reducing depressive and anxiety symptoms. Knowledge scores were significantly higher in the intervention group post-intervention, but not at 3-month follow-up. No significant intervention effects were observed for depressive symptoms. However, the intervention group showed a greater reduction in anxiety symptoms at 3-month follow-up compared to active control. While these findings suggest short-term symptom reduction, longer-term follow-up is needed to assess sustained prevention effects. The intervention was well-received by teachers and students, and no adverse effects were observed.

**Implications of all the available evidence**

Trauma-informed and inclusive co-design with young people is important to improve the effectiveness of universal, school-based interventions. This study shows that such interventions show promise in improving mental health knowledge gain and reducing anxiety symptoms for adolescents. These findings also raise important questions about whether current universal interventions are sufficiently designed to reduce depressive symptoms, or whether enhancements, such as increased dosage, longer follow-up, or more tailored content, are needed to improve efficacy.

## Introduction

Mental disorders are highly prevalent in young people globally and associated with substantial disability.^1^ Alarming increases in internalising conditions, such as depression and anxiety, in recent cohorts of adolescents,^2^ underscore the urgent need for effective and scalable prevention approaches.

Schools represent a promising setting for the delivery of programmes to prevent mental health problems, offering broad reach, developmental appropriateness, and established referral pathways.^3^ Universal programmes, delivered to an entire cohort, have many advantages in terms of ease of delivery, aligning with the preferences of schools and teachers, and minimising the potential for labelling or stigma that is of concern when programmes are targeted to those at greater risk of disorder.^4^^,^^5^ While some conceptual frameworks for universal prevention emphasise the goal of preventing the onset of new symptoms in individuals without pre-existing symptomatology, other frameworks recognise that universal interventions can also yield benefits for individuals with varying levels of existing symptoms. This broader conceptualisation acknowledges that symptom reduction, even among non-clinical populations, can mitigate risk for future disorder. However, the evidence base for universal, school-based interventions to prevent depression and anxiety remains mixed. Meta-analyses have demonstrated that universal mental health programmes can lead to small, short-term reductions in depression and anxiety symptoms compared to control conditions,^6^^,^^7^ while other meta-analyses have found no benefit in reducing symptoms of depression and anxiety.^8^ Moreover, three recent, large randomised controlled trials (RCTs) of universal school-based prevention programmes for depression all reported no significant effects of the intervention on depressive symptoms,^9^, ^10^, ^11^ and in two of these, there was some indication that the interventions had unintended iatrogenic effects.^12^^,^^13^

The *OurFutures Mental Health* programme (previously known as ‘*Climate Schools Mental Health*’) is one such programme showing mixed effects. It was originally trialled between 2013 and 2016 in Year 8/9 students in Australia, as part of a four-arm cluster RCT that assessed the impact of three school-based interventions: *Climate Schools Mental Health, Climate Schools Substance Use*, and a combined version of both, on the prevention of depression, anxiety, and substance use, compared to an active control (standard health education). Over 30-month follow-up, students who received the combined mental health and substance use intervention demonstrated improved knowledge related to alcohol, cannabis, and mental health, alongside a slower increase in anxiety symptoms compared to control.^11^ However, post-hoc analyses indicated that students in the standalone mental health condition, while showing gains in mental health knowledge, did not experience improvements in other mental health outcomes relative to the control group.^12^ Notably, this group also reported temporary increases in internalising symptoms at 6- and 12-month follow-ups, though these effects were not evident at later timepoints.^12^

Due to these effects, the *OurFutures Mental Health* programme underwent a comprehensive adaptation process between 2022 and 2023, detailed extensively elsewhere.^14^ This four-stage, mixed-methods process began with analysis of prior student evaluation data,^15^ which revealed the need to ensure that the content covers wide aspects of mental ill-health, such as trauma and adversity, stigma, and gender and sexuality diversity. Adaption proceeded via focus groups and interviews with adolescents (including lesbian, gay, bisexual, trans, queer, asexual and other identities [LGBTQA+] youth), expert clinical review with particular attention to trauma-informed principles, and ongoing consultation with youth mental health researchers. Key updates included making the programme trauma-informed and LGBTQA+ affirmative, enhancing the relatability and diversity of characters and storylines, and restructuring content delivery to align with the principles of proportionate universalism.^14^ This included the introduction of optional, at-home activities to provide additional support for students seeking more information and skills, while maintaining a low-intensity, universally-delivered core curriculum of evidence-based psychoeducation and cognitive-behavioural skills training. These adaptations were grounded in youth feedback and expert guidance, aiming to improve engagement, relevance, and safety for all students within a trauma-informed approach,^14^ with the provision of psychoeducation and CBT-based skills practice expecting to produce benefits for mental health knowledge, depression, and anxiety.

The current manuscript reports the outcomes of a cluster RCT evaluating the efficacy of the updated *OurFutures Mental Health* programme in improving mental health knowledge and reducing symptoms of depression and anxiety among Year 8/9 students in Australian secondary schools. As per the pre-registered protocol,^16^ our three primary hypotheses were that compared to students in control schools, students in intervention schools would show improved mental health knowledge, reduced depressive symptoms, and reduced anxiety symptoms, at 3-months post-baseline. The inclusion of depressive and anxiety symptoms as primary outcomes was consistent with the intervention's theoretical underpinnings and with prior universal CBT-based trials that have assessed symptom change across the full sample.^10^^,^^11^

We also pre-registered subgroup analyses to examine the effect of the intervention on depression among adolescents who reported elevated depressive symptoms at baseline, and the effect of the intervention on anxiety among adolescents reporting elevated anxiety symptoms at baseline.

## Methods

### Study design

A two-arm cluster RCT was conducted from 2022 to 2024 in 10 secondary schools across the Australian states of New South Wales (NSW), Queensland (QLD), South Australia (SA), and Victoria (VIC). Ethical approved was granted by the University of Sydney Human Research Ethics Committee (approval number 2022/700), and the NSW State Education Research Application Process (SERAP) for approval to conduct research in government schools (state or public) (approval number 2022349). The protocol was prospectively registered with the Australian and New Zealand Clinical Trials Registry (ACTRN12622001582741, https://www.anzctr.org.au/Trial/Registration/TrialReview.aspx?id=384898) and published elsewhere.^16^ The trial adheres to CONSORT guidelines. Two changes were made to the protocol after the trial commenced (but prior to the first participant enrolment). First, eligibility criteria for schools as per the registered protocol was at least 70 Year 8 students enrolled; however, due to difficulties with recruitment and to allow interested schools to participate, this criterion was removed (change made May 5, 2023). Relatedly, recruitment was expanded to include Year 9 students in addition to Year 8 students (approved May 31, 2023).

### Participants

In 2022–2024, 530 independent (non-government) schools across all Australian states and territories, along with 446 government schools in NSW only (due to jurisdictions for ethical approval for government schools) were approached using publicly available contact details and study advertisements were also promoted to the research team's networks. Eligible participants were consenting Year 8 or Year 9 students attending one of the participating schools. Students provided written informed consent, and parental consent was either passive (independent schools) or active (government schools) according to the conditions of ethical approval obtained.

### Randomisation and masking

Schools were randomised at the school level using the *Blockrand* package in R. A biostatistician, independent of school recruitment, performed the randomisation and enrolled schools were allocated to either the intervention or a standard health education programme (active control). Stratification was based on school binary sex recorded at birth composition (either coeducational, predominantly male (>60%), or predominantly female (>60%)). The randomisation sequence and group assignments were stored in secure folders to ensure blinding of investigators and research staff. Due to practical constraints, blinding was not possible for team members directly involved with schools (e.g., research assistants coordinating intervention delivery with teachers).

### Procedures

Schools allocated to the intervention condition implemented the *OurFutures Mental Health* programme between August to December 2023. *OurFutures Mental Health* was designed as a universal, school-based programme grounded in cognitive-behavioural therapy (CBT) and trauma-informed principles. The intervention integrates psychoeducation, coping skills, cognitive restructuring, emotion regulation strategies, problem-solving, and interpersonal skill development. These components are applicable to all students, regardless of baseline symptom levels, and may help buffer against the development or worsening of mental health symptoms. The programme consists of six online lessons delivered over approximately six weeks. Lessons are comprised of a 20-min cartoon storyline that follows a group of Australian adolescents navigating everyday challenges and mental health concerns. The intervention incorporated a blend of LGBTQA+ and non-LGBTQA+ narratives, with a deliberate focus on inclusion through normalisation. Rather than centring storylines around gender and sexuality, LGBTQA+ characters were portrayed in everyday scenarios that reflected common adolescent experiences, such as friendship, academic stress, and family dynamics. This approach was informed by lived experience recommendations that LGBTQA+ young people experience life beyond their identities and should be represented as such.^14^ Additionally, characters and storylines had themes of coping with traumatic events carefully embedded. The cartoons contained embedded quizzes with immediate feedback to reinforce learning of the concepts and CBT skills covered in that lesson. Lessons also included a 20-min teacher-facilitated activity, either completed as a group or individually. These activities reinforced the information in the cartoons by encouraging students to apply cognitive-behavioural strategies to the characters in the cartoon, allowing for further individual or interactive communication between students and group discussion (depending on the format chosen). In addition to the classroom components, students were offered additional optional exercises to complete independently at home, providing the opportunity to apply the CBT skills learnt in class to their personal scenarios in a trauma-informed approach. This enabled a more targeted approach within universal delivery, providing lower-intensity content promoting general wellbeing and socio-emotional skills training to all adolescents, while offering additional skills training, practice, and intervention activities to adolescents opting in for more therapeutic content. Students and teachers were also provided with lesson summaries, which included information on seeking support, relevant services, and evidence-based information regarding trauma among young people and gender and sexuality diversity. Further detail on the content of the intervention has been published previously.^14^^,^^16^ The hypothesised mental health benefits were primarily expected to arise from the delivery of psychoeducation and the CBT-based strategies, which are applicable to a wide range of stressors experienced by adolescents (see Supplementary Figure S1 for programme logic).

After each lesson, teachers completed a logbook to assess adherence and fidelity to the intervention. These logbooks captured whether the students completed the online module in its entirety, which activities were used for that lesson, and any deviations from the prescribed content. Additionally, at the end of the six-week intervention, teachers completed a brief survey evaluating *OurFutures Mental Health*, providing their overall rating, how easy or difficult they found implementation, quality of the classroom activities, and whether they would use the programme in future or recommend it to others. Students also completed a post-intervention survey to measure engagement and acceptability. Measures included students’ overall rating, their rating of the cartoon stories including their relevance and usefulness, and whether they think the content or skills will be helpful for their mental health in future. Uptake of the home activities was captured through the online platform that hosted the intervention.

Control schools implemented their usual health education in 2023–2024, which includes mental health and wellbeing content as mandated in the Australian curriculum. Staff in control schools completed a logbook to record the details of mental health education received by students across the duration of the trial.

### Outcomes

Three pre-registered primary outcomes were mental health knowledge, depressive and anxiety symptoms at 3-months post-baseline. To assess trial outcomes, students completed an online self-report survey at baseline, post-intervention (approximately 6-weeks post-baseline), and at 3-months post-baseline (primary time-point).

Birth sex and gender were self-reported in response to the questions “What sex were you assigned at birth?” (Male/Female/Another Term/Prefer not to answer) and “How do you describe your gender?” (Male/Female/Non-binary/I use a different term, please specify/Prefer not to answer), as aligned with accepted practice for collecting information on gender identity.^17^ Gender identity was transformed to a four-level variable with responses Boy (cisgender and transgender)/Girl (cisgender and transgender)/Gender Diverse (including non-binary)/Prefer not to answer.

A 16-item, intervention-specific assessment was used to evaluate students' understanding of mental health concepts presented during the programme (Supplementary Material). There were four multiple choice questions, and 12 statements requiring students to indicate whether that statement was true or false. They could also indicate don't know, which was included as a response option to minimise the potential for students to guess true or false correctly. A total score was created, reflecting the sum of correct answers.

Depressive symptoms were assessed using the Patient Health Questionnaire for Adolescents (PHQ-A),^18^ a 9-item instrument designed to measure the frequency of depressive symptoms over the past two weeks. For this study, one item assessing suicidal ideation was excluded due to ethical considerations, resulting in an 8-item version with possible scores ranging from 0–24.^18^ Additionally, planned subgroup analyses were conducted for students who met the threshold for moderate depression (scores ≥10) at baseline.^19^

Anxiety symptoms were measured using the 7-item Generalised Anxiety Disorder scale (GAD-7),^20^ which measures the frequency of symptoms of anxiety over the past two weeks. Scores were summed to create a total score ranging from 0 to 21. The GAD-7 is a validated screening tool for generalised anxiety, with scores of 5–9 reflecting mild anxiety, 10–14 indicating moderate anxiety, and 15 or higher reflecting severe anxiety.^20^ Again, subgroup analyses were conducted focussing on students who scored ≥10 at baseline, indicating moderate anxiety.^20^

Both the PHQ-A and GAD-7 have been shown to be valid and reliable measures of depression and anxiety among adolescents.^18^^,^^20^

Secondary outcomes as per the registered protocol included wellbeing, help-seeking, social, emotional, and behavioural problems, psychological distress, functional impairment, quality of life, resource use, and alcohol use. These were not included in the current manuscript to maintain conciseness. These will be reported in a separate publication which will also include the final follow-up (non-primary end point) from the trial in 2026.

### Statistical analyses

As per the pre-registered protocol, intention-to-treat analyses were planned for all primary outcomes, whereby all randomised students were analysed in the groups that they were assigned. Sample size calculations were based on the number of students, using the method developed by Heo and Leon (2009)^21^ for longitudinal cluster-randomised trials, which indicated that six schools per group with approximately 100 students each (N = 1200 students) would provide 80% power to detect a small effect size of 0.15 (α = 0.05) at trial endpoint. The trial was powered to detect an effect size of 0.3 (α = 0.05) in the subgroup analyses with an estimated 396 students exhibiting elevated symptoms. Due to school withdrawals, this sample size was not achieved, with 793 students at baseline: 248 with elevated depression and 202 with elevated anxiety. Post-hoc power analysis was conducted (Supplementary Material) and revealed that the present study was not sufficiently powered to detect estimated effect sizes, with twelve schools (six intervention, six control) required for anxiety and depressive symptoms, compared to the sample size of 10 schools (six intervention, four control) achieved.

Linear mixed effects regression investigated whether the receiving the intervention improved knowledge scores and reduced depression and anxiety scores among the whole sample and for students exceeding the symptom threshold for moderate anxiety (GAD total score ≥10) or depression (PHQ total score ≥10) at baseline. All analyses were performed in R, using the *lme4* package.^22^ To address the nested structure of the data, mixed-effects models were specified with random intercepts for participants and schools. Estimated marginal means were calculated, which adjusted for covariates included in the model, providing a clearer picture of the group-by-time effects independent of confounding variables. To determine the optimal time specification for each outcome, we compared linear, quadratic, and categorical representations of time using linear mixed-effects unconditional models (without covariates) with random intercepts for participants and schools. Model fit was assessed using Akaike Information Criterion (AIC), Bayesian Information Criterion (BIC), and likelihood ratio tests. Fit statistics are presented in Supplementary Table S1. Categorical time was selected for all outcomes based on superior or comparable fit and greater interpretability across discrete measurement waves. As randomisation was stratified by school sex composition, all models adjusted for assigned sex at birth. Models were additionally adjusted for age (to account for developmental differences between Year 8 and Year 9 students). No additional baseline covariates were included, as randomisation is expected to balance confounders across groups. Cluster-level variability was accounted for using random intercepts for schools. All mixed-effects models employed restricted maximum likelihood estimation (REML), which provides unbiased estimates under the assumption that data are missing at random by maximising the likelihood based only on the observed data. To explore the plausibility of this assumption, patterns of missing data were examined by comparing baseline outcomes and potential confounders between participants who completed at least one follow-up and those who did not. Additionally, for each outcome, a binary variable was created to distinguish participants with only baseline data from those with follow-up data. This was used to assess whether attrition differed between groups for each outcome (Supplementary Table S2). Additionally, we conducted sensitivity analyses to examine potential bias due to baseline imbalance and adjusting for baseline scores in each of the outcomes. Results are presented in Appendix A, Appendix A.

### Role of the funding source

The funder of the study had no role in study design, data collection, data analysis, data interpretation, or writing of the report. Authors had full access to the data in the study. All authors had final responsibility for the decision to submit for publication.

## Results

**Fig. 1:**
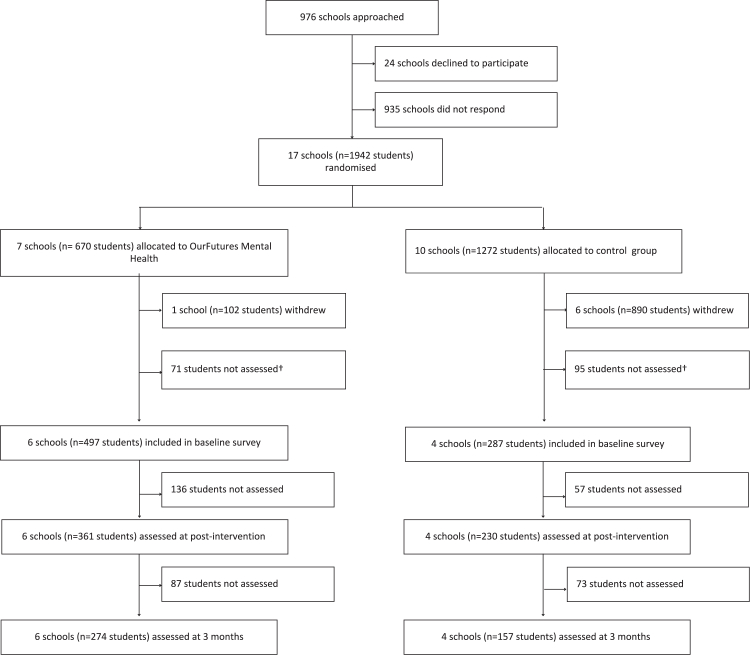
Trial profile.

From 6 December 2022 to 12 April 2024, 17 schools were recruited and randomised (1785 students), with the first school enrolled on 16 February 2023 and the first participant enrolled on 14 July 2023. Seven school withdrew prior to baseline (6 control; 1 intervention). Schools cited several reasons for withdrawal, most commonly, a lack of time to complete the trial activities and concern with the gender and sexuality diversity questions and content covered in the survey and intervention. A total of 784 students from 10 schools (6 schools assigned to the intervention group (497 students) and 4 schools to the control (287 students); Fig. 1) consented and participated in the baseline survey (mean age 13.8 years, SD 0.78, 467 [59.6%] male). Table 1 reports baseline demographics and descriptive statistics for each outcome of interest by intervention group.

**Table 1:** Baseline demographics and descriptive statistics for each outcome of interest by intervention group and timepoint.

	Intervention (n = 497)	Control (n = 287)	
No of schools	6	4	
Age, mean (SD)	13.86 (0.81)	13.70 (0.71)	
Gender, n (%)			
Boy (cisgender and transgender)	359 (72.7)	108 (37.6)	
Girl (cisgender and transgender)	126 (25.5)	168 (58.5)	
Gender diverse (including non-binary)	4 (0.8)	8 (2.8)	
Prefer not to say	5 (1.0)	3 (1.0)	
Knowledge scores Mean (SD)			Cohen's d
Baseline	7.00 (2.86)	6.57 (2.85)	0.15
6-weeks	8.61 (3.39)	6.87 (3.20)	0.52
3 months	8.30 (3.65)	7.58 (3.11)	0.21
Depressive symptoms Mean (SD)			
Baseline	6.62 (5.74)	8.72 (6.77)	−0.33
6-weeks	5.88 (5.60)	8.11 (6.72)	−0.36
3 months	6.27 (5.94)	7.89 (6.63)	−0.26
Anxiety symptoms Mean (SD)			
Baseline	5.56 (5.48)	7.51 (6.52)	−0.33
6-weeks	4.45 (5.23)	7.12 (6.44)	−0.46
3 months	5.35 (5.43)	6.76 (6.16)	−0.24

SD: standard deviation.

Of the baseline sample, 556 participants (71%) had complete data for all primary outcomes on at least one follow-up survey. Supplementary Table S2 reports descriptive statistics and regression tests of baseline characteristics and outcomes by follow-up status and whether there were differences in attrition for each outcome by intervention group. In brief, there were no differences in attrition related to trial group, age, gender, or the primary outcomes, except for knowledge scores for which higher knowledge scores at baseline were weakly associated with a reduction in the odds of attrition (OR = 0.93, 95% CI: 0.88–0.99, p = 0.016).

**Fig. 2:**
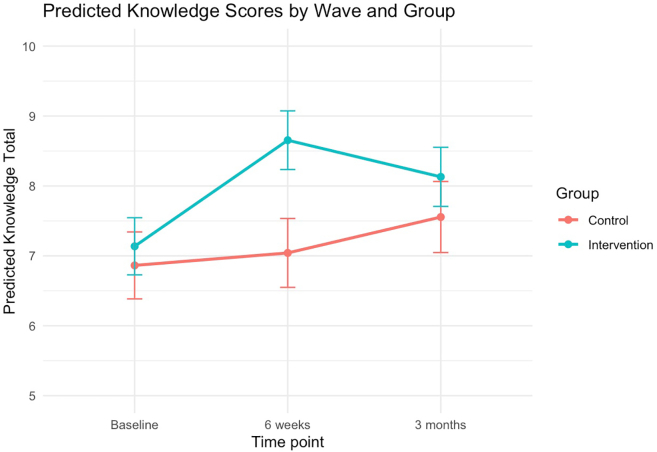
Predicted knowledge scores by wave and group. Estimated marginal means of knowledge across time points for the intervention and control groups. Error bars reflect standard errors. Estimated marginal means at 6 weeks were higher for the intervention group compared to the active control group. This difference was no longer significant at the 3-month follow up.

As shown in Table 2, at the post-intervention timepoint participants who received the intervention had higher knowledge scores than those in the active control group (β = 1.34, 95% CI 0.81–1.86, p < 0.0001). This difference was no longer significant at the 3-month follow up (β = 0.30, 95% CI: −0.32 to 0.91, p = 0.34). However, visual inspection of the model-estimated trajectories (Fig. 2) suggested this may have been influenced by an increase in mental health knowledge in the control group. Thus, within-group, post-hoc analyses were conducted to assess changes in mental health knowledge from baseline to 3-months, using Wilcoxon signed-rank tests due to non-normality of differences. This revealed a significant increase in mental health knowledge for both intervention and control, with a mean increase of 0.68 points (SD = 2.76) in the control group (V = 3704.5, p = 0.0053) and 0.90 points (SD = 3.32) in the intervention group (V = 13405.5, p < 0.0001); not shown.

**Table 2:** Results of mixed effects linear regression models.

Predictor	Knowledge β (95% CI), p	Depression β (95% CI), p	Anxiety β (95% CI), p
Time^a^: 6 weeks	0.18 (−0.24, 0.60), 0.41	−0.88 (−1.54, −0.23), 0.0089	−0.59 (−1.22, 0.03), 0.066
Time^a^: 3 months	0.69 (0.20, 1.19), 0.0063	−0.42 (−1.20, 0.33), 0.28	−0.16 (−0.91, 0.55), 0.67
Intervention	0.27 (−0.94, 1.46), 0.67	0.03 (−1.29, 1.38), 0.97	0.05 (−1.25, 1.37), 0.94
Birth Sex^b^: Male	−0.73 (−1.29, −0.18), 0.011	−5.07 (−6.07, −4.13), <0.0001	−4.94 (−5.86, −4.02), <0.0001
Age	0.49 (0.21, 0.75), 0.0003	0.28 (−0.21, 0.77), 0.26	−0.02 (−0.48, 0.45), 0.93
6wks × OFMH	**1.34 (0.81, 1.86), <0.0001**	0.01 (−0.82, 0.84), 0.99	−0.57 (−1.35, 0.22), 0.16
6wks × OFMH Cohen's d	0.63	0.002	−0.19
3mo × OFMH	0.30 (−0.32, 0.91), 0.34	−0.94 (−1.88, 0.04), 0.055	**−1.05 (−1.93, −0.12), 0.024**
3mo × OFMH Cohen's d	0.14	−0.29	−0.34

CI = Confidence Interval. OFMH = *OurFutures Mental Health*.

Bold indicates significant interaction at p < .05.

a Reference category = baseline.

b Reference category = female.

There was no between group difference in depression at either the post-intervention timepoint (β = 0.01, 95% CI: −0.82–0.84, p = 0.99) or 3 months (β = −0.94, 95% CI: −1.88–0.04, p = 0.055); Table 2. Estimated marginal means showed a pattern of decreasing depression scores over time in both groups, with slightly greater reductions observed in the intervention group at 3 months compared to the active control group, though these differences were not statistically significant (Supplementary Figure S2; Supplementary Table S5).

**Fig. 3:**
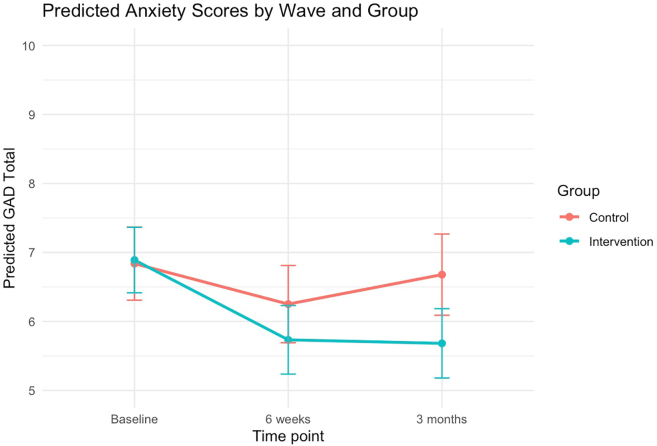
Predicted anxiety scores by wave and group. Estimated marginal means of anxiety across time points for the intervention and control groups. Error bars reflect standard errors. Estimated marginal means show that anxiety scores were lower in the intervention group compared to control at both follow-up timepoints.

There was a statistically significant interaction for anxiety at 3-months, where participants in the intervention showed greater reduction in anxiety compared to active control (β = −1.05, 95% CI: −1.93 to −0.12, p = 0.024); Table 2. The intervention by time interaction at the post-intervention timepoint was not significant (β = −0.57, 95% CI: −1.35 to 0.22, p = 0.16). Estimated marginal means showed that anxiety scores were lower in the intervention group compared to control at both follow-up timepoints (Fig. 3; Supplementary Table S5).

At baseline, almost one third of students reported elevated symptoms of depression (247/774, 31.9%) and one quarter (202/773, 26.1%) reported elevated anxiety. Subgroup analysis (Supplementary Table S6) revealed no significant intervention by time interaction at either the 6-week (β = −1.00, 95% CI: −2.56 to 0.57, p = 0.21) or the 3-month (β = −0.74, 95% CI: −2.52 to 1.07, p = 0.43) follow-up timepoints for students with elevated depressive symptoms. Similarly, there was no significant intervention by time interaction at either the 6-week (β = −1.69, 95% CI: −3.45 to 0.06, p = 0.061) or 3-month (β = −1.31, 95% CI: −3.22 to 0.76, p = 0.204) timepoints for students with elevated anxiety symptoms.

Adherence to the intervention by teachers was generally good, with 75–100% of classes completing the cartoon module in its entirety. The most cited issue for not completing the cartoon modules and activities was a lack of time. Logbooks for control schools (Supplementary Table S7) indicated that three out of the four schools provided mental health education to their Year 8/9 students, with one school spending seven lessons on mental health content and the other two schools spending four lessons on mental health during 2023. Specifically, three schools reported completing content on help seeking, two schools on identifying anxiety and depression, and one school covered activity scheduling, facing fears, assertiveness and responding to stressful situations.

Across the six intervention schools, 139 students and 10 teachers provided feedback about the *OurFutures Mental Health* programme. Student feedback (Supplementary Table S8) indicated that just over half of the sample rated the programme as good or very good (73, 52.5%), found the information in the cartoons helpful (69, 50.7%), felt the skills and information they learnt would help them in the future (69, 51.1% of 135). Those assigned female at birth rated the programme more highly overall and reported greater satisfaction with the storylines compared to those assigned male at birth (Supplementary Table S9). More detail on student feedback can be found in Appendix A, Appendix A, Appendix A, Appendix A, Appendix A, Appendix A, Appendix A, Appendix A, Appendix A, Appendix A, Appendix A, Appendix A, Appendix A, Appendix A, Appendix A, Appendix A, Appendix A, Appendix A, Appendix A, Appendix A, Appendix A, Appendix A, Appendix A, Appendix A, Appendix A, Appendix A, Appendix A, Appendix A, Appendix A, Appendix A, Appendix A, Appendix A, Appendix A. A minority of students from intervention schools (62, 12.5%) engaged with the home activities. Teachers’ overall ratings were generally positive, with 50% of teachers rating the programme as good/very good, and the remaining 50% rating the programme as average. Most teachers rated *OurFutures Mental Health* as better than most programmes (60%), found it easy to implement (70%), and rated the educational quality of the activities highly (90%). Qualitative feedback revealed teachers had some difficulty in covering the content within the time allocated (n = 3), student engagement (n = 6), technical or resourcing issues (n = 3), and understanding the importance of providing representation of transgender experiences (n = 3) included. Further information can be found in Appendix A, Appendix A.

## Discussion

This study evaluated the efficacy of the *OurFutures Mental Health* programme in promoting mental health knowledge and reducing symptoms of depression and anxiety among adolescents. Participants who received the intervention showed significantly higher mental health knowledge immediately post-intervention, although this effect was not sustained at 3 months. No significant intervention effects were observed for depressive symptoms at either timepoint. However, estimated marginal means suggested a pattern of greater reductions in depression scores in the intervention group by the 3-month time point, though these differences did not reach statistical significance. With regards to anxiety symptoms, the intervention group showed a greater reduction in anxiety symptoms at 3-month follow-up compared to the active control group. No significant effects were observed in the subgroup analyses of students with elevated depression or anxiety symptoms at baseline. Importantly, there was no evidence of harm in the overall sample, nor among those with elevated symptoms. These findings suggest the programme may be effective in enhancing short-term mental health literacy and reducing anxiety symptoms, compared to usual health education.

Results of the current trial reflect an improvement from the previous trial of *OurFutures Mental Health,* conducted from 2013–2016.^11^ While an increase in knowledge and a reduction in anxiety symptoms was found when the previous version of the programme was combined with the *Substance Use* module,^11^ post-hoc analyses found these effects were not evident when *OurFutures Mental Health* was delivered alone.^12^ The results of the current study suggest that the updated *OurFutures Mental Health* intervention can be delivered as a standalone intervention to adolescents that may produce short-term increases in mental health literacy and reductions in anxiety symptoms. While improvements in mental health knowledge in the intervention group relative to control were not sustained by our primary timepoint of 3-months, from the model-estimated trajectories (Fig. 2) this may have been influenced by an increase in mental health knowledge in the control group over the trial period. Indeed, post-hoc analyses revealed increases in mental health knowledge from baseline to 3-months for both groups. Teacher logbooks confirm that three out of four control schools received mental health education during the trial, with substantial overlap with the content of the intervention. This potentially limited our ability to find differences between groups. Nevertheless, the intervention demonstrated short-term gains in mental health literacy beyond those seen in control schools. Positive teacher feedback—60% rating the programme better than most, 70% finding it easy to implement, and 90% rating the educational quality highly—further supports the added value of *OurFutures Mental Health*.

There was no evidence for a significant effect of the intervention on depression. This replicates findings from several other recent trials of school-based universal prevention of depression.^9^, ^10^, ^11^ It is unclear why depression appears so difficult to change through universal prevention programmes. Similar to other trials,^13^ approximately one third of our sample had moderate symptoms of depression at baseline; thus, it is possible that these programmes are not being delivered early enough or do not contain enough of a therapeutic dose to shift depressive symptoms in such a large proportion of students who are already coping with these symptoms. However, in contrast to MYRIAD, which found iatrogenic effects for those with elevated symptoms at baseline,^13^ we found no evidence to suggest iatrogenic effects of the intervention on those with elevated symptoms. In contrast, the pattern of model estimated marginal means suggested that depression scores decreased to a somewhat greater degree in the intervention group compared to the control group, although these differences did not reach statistical significance. It is also important to note that the study was underpowered to detect small effects due to a lower-than-planned sample size, which may have limited our ability to detect statistically significant differences in depressive symptoms.

The results of the current study suggest more can be done to improve student enjoyment of the intervention. Responses to the feedback questionnaire indicated about 50% of students felt positively about the programme, 30% of students were ambivalent toward the programme, and 20% did not like the programme. This variability is not unexpected in universal programmes, which must cater to a wide range of experiences and symptom levels. While we affirm the importance of student autonomy, it is worth reflecting on how students perceive other core subjects, such as mathematics, and whether enjoyment alone should determine the value of educational content. Universal interventions play a critical role in the school setting, even for students who may not find them personally relevant. They are essential in supporting more equitable access, cost-effectiveness and scalability, stigma reduction, and improving knowledge of common mental health conditions in the population overall.^5^ Providing accurate, developmentally appropriate information on mental health through schools can help counter misinformation, reduce stigma, and lay the foundation for mental health literacy.^5^ Novel methods to improve students’ enjoyment, such as offering choice through programme design elements like choosing a personal avatar or “chose your own adventure” style storylines are important next steps to boost engagement within universal delivery.

Likewise, teacher feedback suggests areas for improvement. Some teachers had overwhelmingly positive ratings about the programme, while others were less supportive. Teachers reported barriers including time to deliver the content, technical issues such as lagging of the cartoons due to the internet connection, and not enough laptops for each student. Teachers' suggestions for improvement (Supplementary Table S11) included promoting engagement and interactivity within the lessons. Teachers also raised concerns around the gender and sexuality diversity content, questioning whether certain aspects were necessary and noting a disconnect with the school's typical ethos. Indeed, the representation of transgender experiences was cited as a factor for some schools withdrawing prior to baseline and intervention delivery. Education around gender and gender diversity is not commonplace in Australian secondary schools and there are no standardised training and education requirements for school staff to learn about gender and gender diversity. Research highlights that transgender and gender diverse young people report significantly higher prevalence rates of mental ill-health relative to their cisgender peers,^23^ and that a supportive school environment is associated with reductions in emotional problems and depressive symptoms.^24^ Thus, further education and training could be provided to assist teachers with understanding the unique mental health needs and experiences of LGBTQA+ students and to promote inclusive and affirming content in school programme materials. Importantly, the variability in student and teacher ratings of the programme may have contributed to the null effects observed for depression. The implementation challenges such as time and technical issues may have moderated student engagement and, in turn, intervention efficacy. Future studies should consider formally examining implementation factors, such as student enjoyment, teacher support, and fidelity, as potential moderators of programme outcomes.

Despite the intervention being trauma-informed and including information and storylines centred on experiences of trauma and adversity experienced by young people, there was no evidence of any harm in our outcome measures, nor were any adverse events reported. This is an important finding as the field of trauma-aware education grows and educators seek information and resources to support the young people they teach.^25^^,^^26^ Given the prevalence of childhood adversity and its impact on mental health,^27^ *not* incorporating trauma into mental health education is a critical oversight—however, it is crucial that this is supported by evidence-based best practice to avoid doing harm to young people. The results of the current study suggest that sensitively addressing childhood trauma and providing young people with psychoeducation and skills training to mitigate the effects of trauma can be safely incorporated into education with young adolescents.

This study had several limitations. First, the length of follow-up was limited to three months, precluding examination of whether observed effects were sustained, or differences appeared over a longer term. This time point was selected to assess short-term efficacy and feasibility, consistent with prior universal school-based trials,^6^ yet future studies should include a longer follow-up period. The current trial has continued participant follow-up beyond the pre-registered primary timepoint of 3-months post-baseline, and the longer-term effect of the intervention will be reported in future publications. The 2013–2016 trial of the previous version of *OurFutures Mental Health* found intervention effects on anxiety symptoms for up to 30 months post-intervention, highlighting the potential durability of preventive effects. Secondly, participant attrition was high despite this relatively short follow-up period. Although attrition is a common challenge in school-based prevention trials, particularly those involving sensitive content, our rate was higher than anticipated, although comparable to other Australian school-based mental health programmes.^28^ While we examined attrition and assumed data was missing at random, it is possible that some bias was unaccounted for in our analyses. The low participation rate among approached schools and the withdrawal of seven schools post-randomisation limits the generalisability of the findings, as the final sample may not be representative of the broader school population. Relatedly, school withdrawal meant the trial was underpowered, limiting the ability to detect smaller intervention effects as confirmed by post-hoc power analysis. Significant intervention effects were nonetheless observed for anxiety symptoms, highlighting the potential meaningful impact of this finding despite limited statistical power. This low statistical power may particularly have affected our subgroup analyses given these only included between one quarter to one third of the overall sample. Future trials may benefit from a range of adjustments to mitigate school withdrawal, such as inbuilt flexibility for different lesson times, education and training for teachers around the relevance of gender and sexuality diversity for student mental health and bringing schools on board earlier in the research process to foster a sense of collaboration for content design and survey development. However, this must be balanced with the need to preserve the integrity of the trial design and reduce expectancy effects. Additionally, as with many school-based trials, variability in implementation fidelity across schools and classrooms may have influenced outcomes. However, this variability also reflects the realities of school environments, where teachers often require flexibility to adapt content to their unique classroom contexts, highlighting the importance of designing interventions that are both structured and adaptable. Finally, the generalisability of findings may be limited by the specific demographic and geographic characteristics of the participating schools. In the current study, all schools except one were independent, thus future research should aim to include more diverse student populations from predominantly government schools to enhance external validity.

In conclusion, the *OurFutures Mental Health* intervention was found to be more effective than usual health education in reducing symptoms of anxiety at 3-month follow-up and promoting mental health knowledge immediately post-intervention, with no evidence of harm. While no significant effects were demonstrated for depression, the findings support the continued refinement of universal approaches. Despite growing debate around the effectiveness of universal school-based mental health interventions,^5^^,^^29^, ^30^, ^31^, ^32^ our findings suggest that, when thoughtfully designed, universal mental health interventions can benefit students without evidence of harm.

## Contributors

LG led the development of this article. NCN, ELB, MT, LB, and LG conceptualised the study. LG, EVK, SB, LB, ELB, NCN, CC, MT, LAG, and KEC led intervention development. MT, KEC, NCN, CC secured funding for this study. LG was responsible for ethics and governance, overall trial coordination, and supervision of staff with oversight from ELB, NCN, and LB. LG, SB, II, EH, AN were responsible for recruitment of schools and data collection. SO and LG were responsible for data analysis and monitoring, with oversight from ELB. Data were directly accessed and verified by SO and LG. All authors had full access to all the data. All authors contributed to developing protocols for the study and reviewed, edited, and approved the final version of the manuscript. All authors had final responsibility for the decision to submit for publication.

## Data sharing statement

The statistical analysis code and de-identified participant data will be made available to researchers on request to the corresponding author and with appropriate reason when accompanied by study protocol and analysis plan. Data will be shared after the approval of a proposal by a committee of the current research team with a signed data access agreement.

## Declaration of interests

MT and NN are developers of the OurFutures Mental Health programme, which has been licenced to the University of Sydney and is being distributed through the not-for-profit organisation, Our Futures Institute. MT and NN are non-executive director of OurFutures Institute, a joint social enterprise not for profit, established in 2022 to distribute the OurFutures programmes and maximise social well-being. LB, LAG, CC, MT and ELB are currently supported by Australian National Health and Medical Research Council (NHMRC) Investigator Grants and KEC is supported by a University of Sydney Horizon Fellowship. LG, SO, SB, II, EH, AN, and EVK declare no competing interests.
