# Supplementary material for: Efficacy of a school-based, universal prevention programme for depression and anxiety in adolescents (OurFutures Mental Health): a two-arm cluster-randomised controlled trial
Source: eClinicalMedicine. 2025 Nov 28;90:103672. doi: 10.1016/j.eclinm.2025.103672 (PMC12702296; doi:10.1016/j.eclinm.2025.103672)
Supplement: Supplementary Material [file mmc1.pdf]

Supplement to: Efficacy of a school-based, universal prevention programme for depression and anxiety in adolescents (OurFutures Mental Health): a two-arm cluster-randomised controlled trial

Table of Contents

Supplementary Figure 1: Logic Model 2

Supplementary Methods: Trial-specific knowledge scale 3

Supplementary Methods: Post-hoc power analysis 4

Supplementary Table 1. Time specifications. 4

Supplementary Table 2. Attrition analyses. 5

Supplementary Table 3. Results of Twisk sensitivity analysis to account for potential bias due to baseline imbalance. 7

Supplementary Table 4. Results of sensitivity analysis controlling for baseline scores in each of the outcomes. 7

Supplementary Table 5. Estimated marginal means and 95% confidence intervals. 8

Supplementary Figure 2. Estimated marginal means of depression by wave and group 9

Supplementary Table 6. Results of subgroup analyses among students with elevated baseline depression scores (Depression column) and students with elevated anxiety scores (Anxiety column) 10

Supplementary Table 7. Control school logbooks on other mental health education received during the trial period. 11

Supplementary Table 8. Student evaluation survey response rate and results. 13

Supplementary Table 9. Associations between student binary sex recorded at birth and evaluation survey outcomes. 14

Supplementary Table 10. Student evaluation survey responses regarding 'one good thing' about the program. 15

Supplementary Table 11. Student evaluation survey responses regarding 'one bad thing' about the program. 16

Supplementary Table 12. Summary of Teacher Feedback 17

Supplementary Table 13. Module-based Feedback 18

Supplementary Figure 1: Logic Model

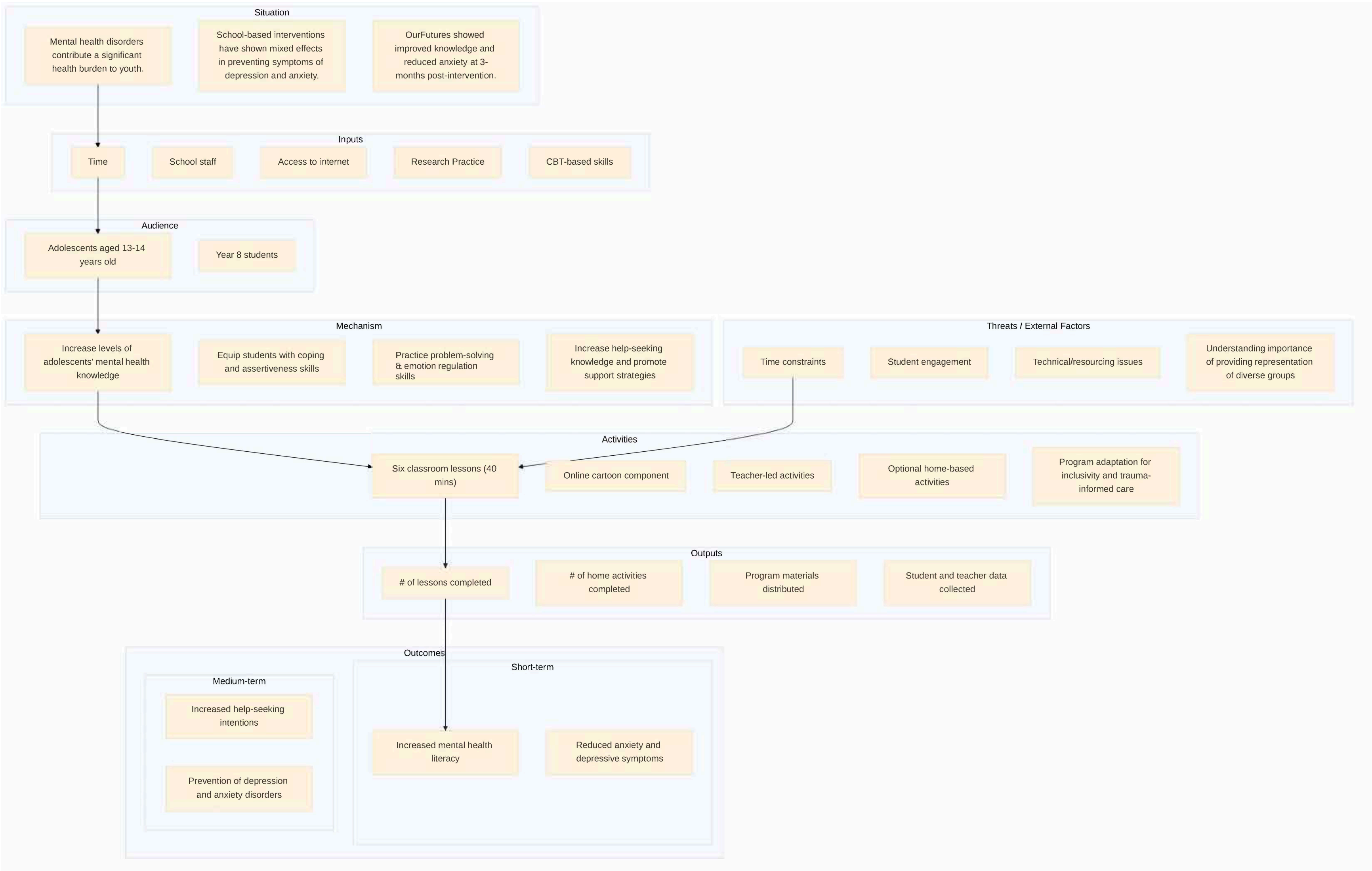

Supplementary Methods: Trial-specific knowledge scale

For each question below, please indicate which response you think is correct. Remember, this is not a test 😊

	True	False	Don't know
**1.** Avoiding things that make you anxious will reduce anxiety long-term.			
**2.** Realistic thinking means that it is most realistic and helpful to make conclusions based on what you first think.			
**3.** Progressive muscle relaxation is an effective strategy for anxiety			
**4.** Being assertive is about making sure things are done your way.			
**5.** A stepladder is a strategy to help you face something you have been avoiding due to anxiety.			
**6.** Only people who have a depressive disorder feel down.			
**7.** The aim of coping strategies is to help manage uncomfortable feelings, not to try to get rid of uncomfortable thoughts and feelings.			
**8.** Anxiety is harmful.			
**9.** Fortune telling, mind reading, and underestimating abilities are all types of thinking traps we can fall into when we are feeling down or anxious.			
**10.** Emotion surfing can help manage any intense emotion.			
**11.** Structured problem-solving is a cure for depressive disorders and anxiety disorders.			
**12.** Thinking we know what other people are thinking is an example of 'fortune telling'			

13. An example of black and white thinking is:
   a. "The weekend is over so it's back to school today"
   b. If I don't get an A on this exam it's a complete failure.
   c. I refuse to do that
   d. Two plus two equals five

14. Activity scheduling helps you to:
   a. Make sure you don't miss out doing things like chores and homework, by setting time aside each day
   b. Limit the time spent on things you enjoy so they don't take up your whole day
   c. Avoid things you don't like doing by using a schedule to fill up your day with positive activities
   d. Make sure you keep doing the things you enjoy and that are important to you, even if low mood or anxiety make you not feel like doing them

15. Controlled breathing is:
   a. A technique to slow your breathing rate that can help reduce anxiety.
   b. A technique that helps to control your mood.
   c. A technique that helps to control your anxiety.
   d. A technique that increases your breath rate and lifts your mood.

16. Which of the following is not a helpful way of handling difficult situations?
   a. Trying to work out what the main problem is, and taking steps to try to solve it
   b. Being hard on yourself in order to improve
   c. Thinking about how you have dealt with similar difficulties in the past
   d. Sharing your concerns with someone you trust

Supplementary Methods: Post-hoc power analysis

Post-hoc power analysis was conducted, with power set to 0·80, correlation among level-1 data set to 0·60, a significance level of 0·05, three time points and the mean number of students at participating schools (n = 79)[18]. Effect sizes of 0·17 and 0·16 were used for depression and anxiety symptoms respectively, consistent with mean effects of a meta-analysis of universal school-based prevention interventions[19]. Post-hoc power analysis revealed that the present study was not sufficiently powered to detect these effect sizes, with twelve schools (six intervention, six control) required for anxiety and depressive symptoms, compared to the sample size of 10 schools (six intervention, four control) achieved.

Supplementary Table 1. Time specifications.

**Outcome**	**Time Specification**	**AIC**	**BIC**
Depression (PHQ)	Linear	9480·6	9507·4
Depression (PHQ)	Categorical	9479·4	9511·5
Depression (PHQ)	Quadratic	9483·2	9515·3
Anxiety (GAD)	Linear	9311·0	9337·8
Anxiety (GAD)	Categorical	9306·0	9338·2
Anxiety (GAD)	Quadratic	9309·8	9342·0
Knowledge	Linear	8019·1	8046·1
Knowledge	Categorical	8000·9	8033·2
Knowledge	Quadratic	8004·7	8037·0

For all outcomes, models specifying time categorically provided the best or comparable fit based on AIC and BIC values. For depression and anxiety, differences between models were small, though categorical time was selected for interpretability. For knowledge, both categorical and quadratic models significantly outperformed the linear model, with categorical time ultimately selected for consistency across outcomes.

Supplementary Table 2. Attrition analyses.

DEPRESSION - PHQ

Characteristic	**1+ Follow-ups (558)**	**0 Follow-ups (N= 226)**	**Odds Ratio**	**p**
**Intervention group**				
Control (N = 287)	203 (70·7%)	84 (29·3%)	--	
OF Mental Health (N = 497)	355 (71·4%)	142 (28·6%)	0·81 (0·33, 2·00)	0·652
**Gender**				
Boy (cis and trans) (N = 467)	313 (67·0%)	154 (33·0%)	--	
Girl (cis and trans) (N = 294)	227 (77·2%)	67 (22·8%)	0·98 (0·61, 1·57)	0·931
Gender diverse (including non-binary) (N = 12)	9 (75·0%)	3 (25·0%)	1·12 (0·28, 4·57)	0·871
Prefer not say (N = 8)	6 (75·0%)	2 (25·0%)	1·22 (0·22, 6·77)	0·823
**Age (Mean (SD))**	13·78 (0·78)	13·86 (0·78)	0·97 (0·78, 1·20)	0·776
**Baseline depression scores (M(SD))**	7·67 (6·20)	6·68 (6·21)	0·99 (0·96, 1·02)	0·401

ANXIETY - GAD

Characteristic	**1+ Follow-ups (N=556)**	**0 Follow-ups (N= 228)**	**Odds Ratio**	**p**
**Intervention group**				
Control (N = 287)	201 (70·0%)	86 (30·0%)	--	
OF Mental Health (N = 497)	355 (71·4%)	142 (28·6%)	0·77 (0·31, 1·90)	0·573
**Gender**				
Boy (cis and trans) (N = 467)	313 (67·0%)	154 (33·0%)	--	
Girl (cis and trans) (N = 294)	225 (76·5%)	69 (23·5%)	1·03 (0·65, 1·65)	0·890
Gender diverse (including non-binary) (N = 12)	9 (75·0%)	3 (25·0%)	1·15 (0·28, 4·71)	0·841
Prefer not say (N = 8)	6 (75·0%)	2 (25·0%)	1·22 (0·22, 6·83)	0·818
**Age (Mean (SD))**	13·78 (0·78)	13·86 (0·78)	0·97 (0·78, 1·20)	0·748
**Baseline anxiety scores (M(SD))**	6·50 (5·95)	5·71 (5·95)	0·99 (0·96, 1·02)	0·596

KNOWLEDGE - TOTAL

Characteristic	**1+ Follow-ups (591)**	**0 Follow-ups (N= 193)**	**Odds Ratio**	**p**
**Intervention group**				
Control (N = 287)	213 (74·2%)	74 (25·8%)	--	
OF Mental Health (N = 497)	378 (76·1%)	119 (23·9%)	0·80 (0·36, 1·78)	0·580
**Gender**				
Boy (cis and trans) (N = 467)	339 (72·6%)	128 (27·4%)	--	
Girl (cis and trans) (N = 294)	232 (78·9%)	62 (21·1%)	1·00 (0·61, 1·62)	0·988
Gender diverse (including non-binary) (N = 12)	11 (91·7%)	1 (8·3%)	0·34 (0·04, 2·73)	0·308
Prefer not say (N = 8)	6 (75·0%)	2 (25·0%)	1·34 (0·24, 7·41)	0·735
**Age (Mean (SD))**	13·79 (0·78)	13·85 (0·79)	0·98 (0·78, 1·22)	0·831
**Baseline knowledge scores**	7·01 (2·77)	6·33 (3·08)	0·93 (0·88, 0·99)	0·016

We noted some crude differences in outcomes at baseline between groups. As a sensitivity analysis to account for potential bias due to baseline imbalance and reduce the risk of our findings reflecting a regression to the mean, we applied an analytic approach described by Twisk et al. (2018). Specifically, we removed the main effect of condition from the mixed effects regression model. As shown in Supplementary Table 3, this adjustment slightly attenuated the intervention effects, though they remained statistically significant.

Supplementary Table 3. Results of Twisk sensitivity analysis to account for potential bias due to baseline imbalance.

**Predictor**	**Knowledge β (95% CI), p**	**Depression β (95% CI), p**	**Anxiety β (95% CI), p**
Time[a]: 6 weeks	0·16 (-0·25, 0·58), 0·436	-0·87 (-1·51, -0·21), 0·009	-0·58 (-1·19, 0·04), 0·065
Time[a]: 3 months	0·68 (0·19, 1·17), 0·007	-0·42 (-1·17, 0·353), 0·284	-0·16 (-0·89, 0·56), 0·657
Birth Sex[b]: Male	-0·69 (-1·25, -0·14), 0·016	-5·00 (-5.96, -4·01), <0·001	-4·80 (-5·71, -3.89), <0·001
Age	0·50 (0·23, 0·76), <0·001	0·23 (-0·26, 0·72), 0·354	-0·07 (-0·54, 0·39), 0·760
6wks × OFMH	**1·35 (0·83, 1·87), <0·001**	0·02 (-0·79, 0·83), 0·963	-0·55 (-1·32, 0·21), 0·162
3mo × OFMH	0·26 (-0·35, 0·86), 0·409	-0·90 (-1·83, 0·04), 0·061	**-1·00 (-1·88, -0·11), 0·027**

[a]: reference category = baseline; [b]: reference category = female. CI = Confidence Interval. OFMH = *OurFutures Mental Health*

Supplementary Table 4. Results of sensitivity analysis controlling for baseline scores in each of the outcomes.

**Predictor**	**Knowledge β (95% CI), p**	**Depression β (95% CI), p**	**Anxiety β (95% CI), p**
Time[a]: 6 weeks	0·17 (-0·21, 0·54), 0·387	-0·91 (-1·48, -0·33), 0·002	-0·63 (-1·18, -0·09), 0·024
Time[a]: 3 months	0·66 (0·23, 1·10), 0·003	-0·33 (-0·99, 0·33), 0·331	-0·07 (-0·72, 0·52), 0·825
Intervention	0·24 (-0·41, 0·89), 0.503	-0·18 (-0·67, 0·31), 0·472	-0·17 (-0·67, 0·24), 0·547
Birth Sex[b]: Male	-0·01 (-0.35, 0·30), 0·943	-0·74 (-1·15, -0·32), <0·001	-0·51 (-0·921, -0·14), 0·029
Age	0·16 (0·003, 0·31), 0·038	0·19 (-0·04, 0·42), 0·099	0·14 (-0·08, 0·35), 0·225
Baseline knowledge scores	0·81 (0·78, 0·85), <0·001	0·82 (0·79, 0·85), <0·001	0.82 (0.79, 0.85), <0.001
6wks × OFMH	**1·33 (0·86, 1·80), <0·001**	0·02 (-0·70, 0·75), 0·948	-0·54 (-1·23, 0·14), 0·122
3mo × OFMH	0·17 (-0·38, 0·71), 0·549	**-0·97 (-1·80, -0·15), 0·022**	**-1·10 (-1·83, -0·28), 0·006**

[a]: reference category = baseline; [b]: reference category = female. CI = Confidence Interval. OFMH = *OurFutures Mental Health*

Supplementary Table 5. Estimated marginal means and 95% confidence intervals.

	Control		Intervention	
	EMM	95% CI	EMM	95% CI
Knowledge				
Baseline	6·86	5·75-7·98	7·14	6·21-8·07
6 weeks	7·04	5·92-8·17	8·65	7·71-9·60
3 months	7·56	6·42-8·69	8·13	7·19-9·07
Depression				
Baseline	8·09	6·80-9·39	8·12	6·97-9·27
6 weeks	7·22	5·90-8·53	7·25	6·08-8·41
3 months	7·67	6·33-9·01	6·76	5·60-7·92
Anxiety				
Baseline	6·84	5·57-8·10	6·89	5·78-8·00
6 weeks	6·25	4·97-7·53	5·73	4·60-6·86
3 months	6·68	5·38-7·98	5·68	4·56-6·81

Supplementary Figure 2. Estimated marginal means of depression by wave and group

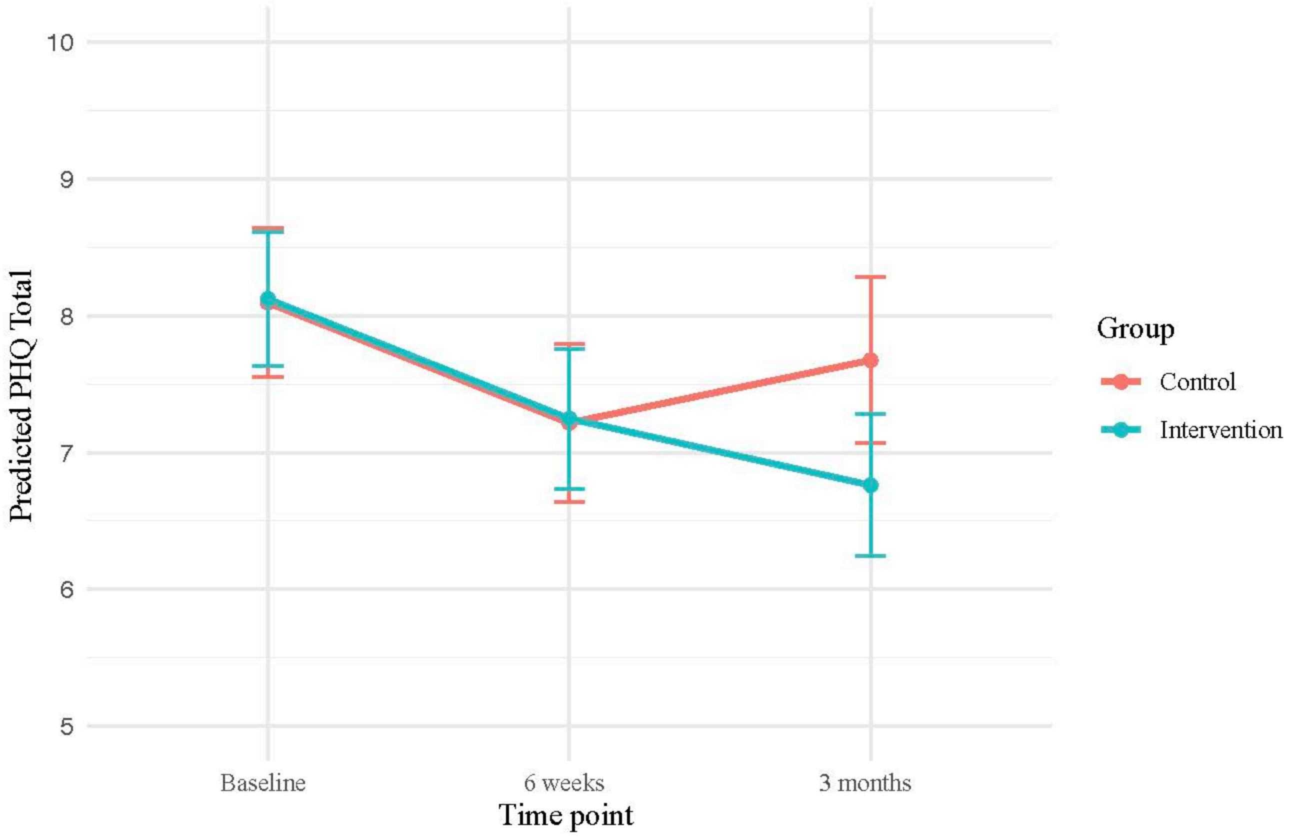

Estimated marginal means of depression across time points for the intervention and control groups. Error bars reflect standard errors. Estimated marginal means show a pattern of decreasing depression scores over time in both groups, with slightly greater reductions observed in the intervention group at 3 months compared to the active control group, though these differences were not statistically significant.

Supplementary Table 6. Results of subgroup analyses among students with elevated baseline depression scores (Depression column) and students with elevated anxiety scores (Anxiety column).

**Predictor**	**Depression**<br>**β (95% CI), p**	**Anxiety**<br>**β (95% CI), p**
Time[a]: 6 weeks	-2·43 (-3·58, -1·29), <0·0001	-2·38 (-3·62, -1·15), <0·0001
Time[a]: 3 months	-3·08 (-4·49, -1·68), <0·0001	-3·00 (-4·61, -1·50), <0·0001
Intervention	-0·41 (-1·68, 0·86), 0·53	-0·03 (-1·45, 1·23), 0·98
Birth Sex[b]: Male	-3·82 (-5·03, -2·61), <0·0001	-2·91 (-4·27, -1·64), 0·0004
Age	0·78 (0·08, 1·48), 0·031	-0·02 (-0·74, 0·73), 0·95
6wks × OFMH	-1·00 (-2·56, 0·57), 0·21	-1·69 (-3·45, 0·06), 0·061
3mo × OFMH	-0·74 (-2·54, 1·07), 0·43	-1·31 (-3·22, 0·76), 0·204

[a]: reference category = baseline; [b]: reference category = female. CI = Confidence Interval. OFMH = *OurFutures Mental Health*

Supplementary Table 7. Control school logbooks on other mental health education received during the trial period.

School	1	2	3	4
Did these students receive any other health education in any of the following areas in 2023? (choice=Identifying anxiety and depression)	Yes	No	No	Yes
Did these students receive any other health education in any of the following areas in 2023? (choice=Realistic thinking and how these skills can positively influence mental health)	No	No	No	No
Did these students receive any other health education in any of the following areas in 2023? (choice=Activity scheduling and facing fears)	Yes	No	No	No
Did these students receive any other health education in any of the following areas in 2023? (choice=Assertiveness and responding to stressful situations)	Yes	No	No	No
Did these students receive any other health education in any of the following areas in 2023? (choice=Structured problem solving and behavioural experiments)	No	No	No	No
Did these students receive any other health education in any of the following areas in 2023? (choice=Help seeking)	Yes	No	Yes	Yes
Did these students receive any other health education in any of the following areas in 2023? (choice=No, students did not receive any other health education in the above area (please skip to the end of the survey))	No	Yes	No	No
How many lessons did you spend on identifying anxiety and depression in 2023?	3			2
How many lessons did you spend on realistic thinking and how these skills can positively influence mental health in 2023?				
How many lessons did you spend on activity scheduling and facing fears in 2023?	0			
How many lessons did you spend on assertiveness and responding to stressful situations in 2023?	2			
How many lessons did you spend on structured problem solving and behavioural experiments in 2023?				
How many lessons did you spend on help seeking in 2023?	2		4	2
What were the main content areas of the lessons about identifying anxiety and depression? (e.g., coping strategies for anxiety and depression, how to support a friend, where to get help, etc)	Coping strategies for anxiety and depression, how to support a friend, where to get help			signs, symptoms, causes, where to get help, how to help a friend
What were the main content areas of the lessons about activity scheduling and facing fears? (e.g., coping strategies for anxiety and depression, how to support a friend, where to get help, etc)	coping strategies for anxiety and depression, how to support a friend, where to get help, etc)			
What were the main content areas of the lessons about assertiveness and responding to stressful situations? (e.g., coping strategies for anxiety and depression, how to support a friend, where to get help, etc)	Seeking support, recognising emotions, defining and recognising stress			
What were the main content areas of the lessons about help seeking? (e.g., coping strategies for anxiety and depression, how to support a friend, where to get help, etc)	Where to go? Links - people - services		Where to get help - personal and	coping strategies, where to get help, how

			community connections.	to support a friend, how to recognise the signs
Identifying anxiety and depression (choice=Traditional)	Yes	No	No	Yes
Identifying anxiety and depression (choice=Internet-based)	Yes	No	No	No
Realistic thinking and how these skills can positively influence mental health (choice=Traditional)	No	No	No	No
Realistic thinking and how these skills can positively influence mental health (choice=Internet-based)	No	No	No	No
Activity scheduling and facing fears (choice=Traditional)	Yes	No	No	No
Activity scheduling and facing fears (choice=Internet-based)	Yes	No	No	No
Assertiveness and responding to stressful situations (choice=Traditional)	Yes	No	No	No
Assertiveness and responding to stressful situations (choice=Internet-based)	Yes	No	No	No
Structured problem solving and behavioural experiments (choice=Traditional)	No	No	No	No
Structured problem solving and behavioural experiments (choice=Internet-based)	No	No	No	No
Help seeking (choice=Traditional)	Yes	No	Yes	Yes
Help seeking (choice=Internet-based)	Yes	No	No	No
When did you deliver the additional health education lesson about identifying anxiety and depression? (month and term)	Term 1 - February to April			term 2 May
When did you deliver the additional health education lesson about activity scheduling and facing fears? (month and term)	Term 1 - February to April			
When did you deliver the additional health education lesson about assertiveness and responding to stressful situations? (month and term)	Term 1 - February to April			
When did you deliver the additional health education lesson about help seeking? (month and term)	Term 1 - February to April		September Term 3, December Term 4.	term 2 May
Did you use any specific programs for mental health education in 2023?E.g. Mind Your Mate	Yes	No	No	No
What was the name of this/these programs? Please list all programs that were used.	Mind Matters - Titan Workbook			

Supplementary Table 8. Student evaluation survey response rate and results.

	1 (Most positive) N (%)	2 N (%)	3 N (%)	4 N (%)	5 (Most negative) N (%)
Overall rating (1 = very good, 5 = very poor)	26 (18·7)	47 (33·8)	42 (30·2)	9 (6·5)	15 (10·8)
Enjoyment of cartoons (1 = liked a lot, 5 = disliked a lot)	30 (21·9)	27 (19·7)	36 (26·3)	20 (14·6)	24 (17·5)
Story satisfaction (1 = liked a lot, 5 = disliked a lot)	28 (20·4)	23 (16·8)	47 (34·3)	14 (10·2)	25 (18·2)
Story relevance (1 = completely relevant, 5 = completely irrelevant)	13 (9·5)	32 (23·4)	29 (21·2)	31 (22·6)	32 (23·4)
Information helpfulness (1 = extremely helpful, 5 = extremely unhelpful)	13 (9·6)	56 (41·2)	33 (24·3)	13 (9·6)	21 (15·4)
Class activity rating (1 = very good, 5 = very poor)	14 (10·3)	46 (33·8)	39 (28·7)	19 (14·0)	18 (13·2)
Likelihood of using skills and information in own life (1 = very likely, 5 = very unlikely)	6 (4·4)	38 (27·9)	50 (36·8)	23 (16·9)	19 (14·0)
Skills and information future effectiveness (1 = help a great deal, 2 = help somewhat, 3 = not at all, 4 = unsure)	17 (12·6)	52 (38·5)	28 (20·7)	38 (28·1)	
Recommend to friends (1 = yes, 2 = no, 3 = maybe)	23 (16·9)	56 (41·2)	57 (41·9)		

Supplementary Table 9. Associations between student binary sex recorded at birth and evaluation survey outcomes.

	Male Mean(SD)	Female Mean(SD)	Mean Difference (Male – Female)	p-value (95% CI)
Overall rating	3·00(0·17)	2·25(0·12)	0·75	<·001 (0·342, 1·158)
Enjoyment of cartoons	3·30(0·20)	2·26(0·15)	0·67	·008 (0·175, 1·172)
Story satisfaction	3·46(0·19)	2·60(0·14)	0·86	<·001 (0·390, 1·332)
Story relevance	3·70(0·19)	3·05(0·14)	0·65	·007 (0·184, 1·112)
Information helpfulness	3·17(0·18)	2·58(0·13)	0·59	·008 (0·157, 1·025)
Class activity rating	3·24(0·17)	2·68(0·13)	0·56	·011 (0·132, 0·989)
Likelihood of using skills and information in own life	3·37(0·16)	2·94(0·12)	0·43	·030 (0·042, 0·816)
Skills and information future effectiveness	2·80(0·15)	2·58(0·11)	0·22	·244 (-0·153, 0·595)
Recommend to friends	2·13(0·11)	2·29(0·08)	-0·16	·249 (-0·421, 0·110)

Note. CI = Confidence Interval. A lower mean represents a more positive score, except for recommending to friends, where a higher mean represents a more positive score.

Supplementary Table 10. Student evaluation survey responses regarding 'one good thing' about the program.

Theme	Sub-theme	Example quotes
Cartoon-based format was enjoyable and engaging	Format including quiz questions and group activities facilitated interest	• "the questions at the end to revise what we learned in the program"<br>• "I liked how the questions correlated with what was going on in the cartoon."<br>• "Could work together as a group on the modules."<br>• "the pop quiz at the end of each lesson"
	Cartoon visuals were interactive and enjoyable	• "I really liked that it included images it helped me stay engaged in every lesson."<br>• "It provided a good visual representation which was easier to gage."<br>• "the cartoons are very helpful and help young adults learn in a fun way."<br>• "It was fun!"
	Timing and length were acceptable, and structure was easy to understand	• "Easy to follow, not hard to read"<br>• "it didn't take that long to do."<br>• "It made sense."
Stories and characters were relatable, realistic, and engaging	Stories were entertaining	• "I mean the story is very good, and entertaining"<br>• "The stories were funny"<br>• "The drama"<br>• "Some funny lines made the lessons more enjoyable and less boring."
	Characters and relationships were inclusive of topics relevant to young people	• "The relevance of the stories."<br>• "Trying to understand the struggles of the age group and what people their age are going through."<br>• "I liked the why they incorporated problems teenagers might be having into cartoons."<br>• "i liked the story and how relevant it is to real life."
	Stories or characters were relatable	• "I liked that they used people at a similar age as me and that they had some similar feelings."<br>• "[name]'s character felt relatable"<br>• "It was pretty relatable"
Learning helpful mental health information and skills	Psychoeducation and learning general mental health information	• "good advice for relevant things"<br>• "The good thing is that they talk a lot about mental health."<br>• "it helps people understand mental health better and also helps them understand them self"<br>• "They teach people how to manage some of their emotions."
	Enhancing coping skills and learning practical strategies to facilitate problem-solving behaviours	• "One benefit of the program is its multitude of strategies to assist with various well-being and mental health problems."<br>• "Good techniques to help with mental health"<br>• It teaches us strategies that could help in hard times"<br>• "It explained the methods very easily so it was easy to understand and use them myself"<br>• "The OurFutures program gives ways of coping with feeling down and/ or upset…."
	Increasing awareness of prevalence of mental health difficulties or other topics	• "It helps me have an idea of what others may be going through."<br>• "good job for bringing attention to mental health"<br>• "i loved the representation i think its really cool to have queer relationships and stuff"<br>• "Opening talking about sensitive topics..."

Supplementary Table 11. Student evaluation survey responses regarding 'one bad thing' about the program.

Theme	Sub-theme	Example quotes
Cartoon-based format was not engaging or enjoyable	Cartoons were too lengthy or featured too much text	• "The cartoons were a bit long."<br>• "There was a lot to read."<br>• "the text was really long sometimes"<br>• "…you should add a feature so you can listen to it instead of reading it"
	Animation format was not engaging	• "to cartoony"<br>• "…maybe next time do photos of young kids not animated"<br>• "not a good way of teaching this kinda thing…i would prefer talking about it not reading fake scenarios."<br>• "it was a little annoying clicking the next page so much next time id prefer just a video then the questions."
	Quiz questions did not facilitate engagement	• "the answers to some of the questions…could have been multiple choice"<br>• "Some of the end questions in the slideshows, I wasn't to keen on"
Characters and storylines were not engaging or relatable enough	Stories were repetitive or boring	• "It was a bit boring"<br>• "it needs more drama"<br>• "How repetitive it was"<br>• "the story is kind cheesy"
	Stories or characters were not relatable or realistic	• "not all related to me which is understandable because everyone is different"<br>• "was a bit unrealistic overall (exaggerated)"<br>• "a bit cringe, the way that the characters talk to eachother isnt the same as real life"<br>• "Characters were difficult to relate to."
	Stories were confusing or difficult to understand	• "Somewhat confusing"<br>• "[Narrators] were intervening in the talk while the characters were talking."
Educational elements were irrelevant or covered difficult content	Content was heavy or uncomfortable	• "some of the information was a bit heavy to handle and very full on."<br>• "One bad thing about the OurFutures program is that some questions may have made some people uncomfortable. I think warning people about the content of the questions before starting/signing up for the program would have been better."
	Over-emphasis on particular topics	• "had to much stuff about sexuality and was very slow to get through"

Supplementary Table 12. Summary of Teacher Feedback

Questions	Teacher Data
Could you please list any ways in which you think the OurFutures program could be improved for the future?	"We did have some issues with a few of our students not being able to log on. Maybe some more interaction with the students during the cartoon stories rather than just at the end?"<br><br>"The class discussion lessons were great and allowed for some deep conversation however on occasion we weren't able to complete all discussion points because of the depth of the conversation. Lessons need to be more than 45mins long."<br><br>"The survey students completed firstly was very confusing for the age range."<br><br>"The modules did not need to include the topic of pronouns as an issue. It caused a lot of controversy for students and parents."<br><br>"We used a double lesson to complete each weeks session. It would have been handy to have more class time to complete other student activities."<br><br>"Making the cartoons more interactive. - different possible scenarios - open speechbubbles - possibility to voice record the dialogs - have audioas for people with reading difficulties"<br><br>"As previously stated in all the other surveys. The cartoon needs to be an mp4 animation. Options need to be in place for diversity of activities that can be implemented according to what time classes have available, eg 10, 15, 20 & 30 min activities."<br><br>"More engaging activities as the students never looked forward to these lessons."<br><br>"The main issue we had was with engagement with the cartoons. When my colleague and I took turns reading out the speech bubbles in an animated way the engagement was high. In other lessons when this did not happen (with the usual classroom teacher) and the students were expected to read the cartoon themselves, or it was rushed through by one person only (not different people fo the different character roles). If the cartoon was animated as a video then engagement would have been much higher and easier. Or if more staff were allocated (at least 2 per class) to read out the different character roles."<br><br>"This is only relevant due to our reduced timeframe, but having this program over a longer span would be great. Incorporating more role-play activities as an option in the classroom (my class LOVE drama). Having a print-out version of all cartoon strips (like a booklet we could keep in the classroom). Increased personalisation (changing the name/hair colour/etc of characters can really improve engagement - not super relevant for my class but could see this working in other contexts). 'Padlet' style activities online – collaborative"
Please list any additional comments you have regarding the OurFutures modules	"I only took the last few classes and helped the students that missed classes to catch up and keep up to date. So my comments are from looking how the program ran with several different teachers taking the classes. Like any lesson plan, the quality of the program and how the students engaged was also a reflection of who was overseeing the lesson content."<br><br>"Great teacher resources. Online platform easy to move through. Very easy to hand over to another teacher without background information."<br><br>"Not recommended for Year 8 students."<br><br>"The class enjoyed the unit, and it encouraged discussion of the issues covered. I found it easy to implement."<br><br>"Y.7/8 liked the format of the cartoons Y.9/10 thought it was too 'try hard' and not natural conversations."<br><br>"Thanks for your efforts. Sorry it didn't work well for our students."

	"I think the program and the skills it teaches are really good and helpful for students. The concern was around engagement (see above) and I think this unfortunately was the main barrier to students accessing the information meaningfully. If the cartoon were presented as an animated video with the characters moving and talking it would be much more engaging."<br><br>"I really loved this program and wish we had more time to do this more thoroughly and without needing to rush towards the end. I also feel that we would have had much better engagement if we had enough devices for each student - many times students needed to share a device which a) reduces the 'completeness' of their online profile and b) reduces the effectiveness of the program as they are less likely to take things seriously when someone else is working with them. Thank you for all your hard work and support, and for creating such a beautiful and important program!"

Supplementary Table 13. Module-based Feedback

Questions	Module 1 (n = 12)	Module 2 (n = 12)	Module 3 (n = 10)	Module 4 (n = 9)	Module 5 (n = 9)	Module 6 (n = 9)
Did your students complete the online OurFutures cartoon component for this module?	100% said yes	92% said yes	100% said yes	100% said yes	100% said yes	100% said yes
Did your students complete the whole online cartoon for this module?	75% said yes	75% said yes	90% said yes	100% said yes	78% said yes	78% said yes
If not, please give reason(s) why	"Many students started the Module 1 cartoon but just a few completed it."<br><br>"Students advised completion of the cartoon and questions. Class moved onto whole class activities. Only on revisiting the student progress did I notice that two students hadn't finished."<br><br>"We have limited laptops at school, some needed to share. These students were slower going through the material."	"A few students started working on module 2, but yesterday I made sure they worked on the module worksheet and completed the case survey."<br><br>"Some did not have enough time"<br><br>"Computer was lagging for all students - were not able to move on - was very slow and could not complete the whole module."	"Students away with illness."	N/A	"Students absent from class."<br><br>"Not engaging. Students were tired of the format."	"not enough time"<br><br>"I believe some students skipped slides towards the end (running out of time)"
Did you go through the student summary with your class for	67% said yes	83% said yes	90% said yes	88% said yes	100% said yes	56% said yes

this module, or instruct them to download it?						
On average, how would you rate the level of engagement of your students with this module?	67% said somewhat or very engaged<br>8.3% said somewhat disengaged	50% said somewhat or very engaged<br>25% said disengaged	60% said somewhat or very engaged<br>40% said disengaged	56% said somewhat or very engaged<br>22% said very disengaged	44% said very or somewhat engaged<br>33% said disengaged	44% said very or somewhat engaged<br>33% said disengaged
Did your students complete the classroom activity or activities?	42% completed all activities<br>50% completed part of the activities<br>8.3% did not complete any activities<br><br>*Understanding emotions*<br>33% chose this activity<br>*Class discussion*<br>33% chose this activity<br><br>None set other activity as homework	25% completed all activities<br>50% completed part of the activities<br>25% did not complete any activities<br><br>*Tackling negative thinking*<br>50% chose this activity<br>*Realistic thinking*<br>8.3% chose this activity<br><br>44% set other activity as homework	20% completed all activities<br>60% completed part of the activities<br>20% did not complete any activities<br><br>*Activity scheduling*<br>50% chose this activity<br>*Stepladder*<br>10% chose this activity<br><br>38% set other activity as homework	33% completed all activities<br>56% completed part of the activities<br>11% did not complete any activities<br><br>*"I" statements*<br>44% chose this activity<br>*Coping strategies*<br>22% chose this activity<br><br>50% set other activity as homework	*Problem solving*<br>44% said yes<br>Of the 56% who said no, none set this as homework	11% completed all activities<br>56% completed part of the activities<br>33% did not complete any activities<br><br>*Thinking traps*<br>30% chose this activity<br>*Coping strategies*<br>30% chose this activity<br><br>38% set other activity as homework
Did you encourage or remind students to use the OurFutures program outside of class?	42% said yes	50% said yes	50% said yes	56% said yes	33% said yes	44% said yes
If yes, how did you encourage them?	"Discussion in class time."<br><br>"I reminded them to read through the information at home and when a situation came up where they might need it."<br><br>"I showed the students how to access the additional tasks and explained that they are optional, some weeks may feel more relevant than others so they can access what they want/need."	"Class discussion at end of lesson to ensure all work was completed."<br><br>"Verbally" (x2)<br><br>"Encouraged them to use the skills we learned today in the lesson in their own lives"<br><br>"We talked about the home activities as a way to engage further if the content is something they resonate with or are finding valuable. Some accessed these	"Reminded students that it they wish to explore deeper these topics they can interact with the optional at home activities"<br><br>"Class discussion at end of lesson. Spoke to any individuals who missed the lesson and asked them to complete the work."<br><br>"Asked them to finish their work as homework"	"By reminding them that they could interact with these modules outside of the class. I also highlighted how this could be beneficial for them."<br><br>"Restorative practices"<br><br>"Class discussion at end of lesson to remind students to complete all work."<br><br>"Verbally"	"Reminded students at the end of the lesson to engage with the option activities."<br><br>"Class reminder at end of lesson and over next few days to ensure work was completed."<br><br>"Verbally."	"Verbally in class with a reminder sent via email to the students and their parents. Students who did not finish set work were given supervised time in their lunch hour to complete."<br><br>"Reminder at end of lesson to complete all work. Also reminded in care group over the next few days to complete work."

	"Verbally" (x2)	during classtime if they finished early."	"We went over how the skills identified in this lesson can be used in the everyday situations."<br><br>"Printed copies were taken home to finish off and students were directed to the home activites (some started these in class)"	"Reminders about the available extras if needed/wanted."		"Verbally"<br><br>"Due to lack of time, we rushed this lesson. I let students know that they would still be able to log in an access content over the holidays so they have been encouraged to access whatever they need to, review, reread, do extra activities, etc."
Do you have any comments / concerns about this module?	"Next time I will encourage them to use the program outside the class time."<br><br>"I am wondering why gender identity needed to be included. There was no lead up discussion to gender identity issues so that was confronting for Year 8's. I found the quiz at the end to be bias, 2 of the 5 questions had a gender identity focus which was not reflective of the number of slides focussing on gender identity."<br><br>"Not with Module 1."<br><br>"A lot of text. Hard to fit it all in one lesson. We did it as a whole class and role played the scenes top make it more interactive and interesting. Year 9/10 thought it was a bit cringy. Year 7/8 were ok."<br><br>"THE CARTOON TAKES TOO LONG, RELIES ON GOOD INTERNET ACCESS (OURS KEPT DROPPING OUT) AND IS TOO WORDY FOR THE STUDENTS."	"We are behind in delivering the programme. This Wednesday, I was away, so I could not teach the class, but I left all the instructions to my colleague. I asked my colleague to ensure all the students completed the base survey and all the activities in Module 1 before moving to Module 2. However, some students could not log back into the 'Our Futures' website; this is the second time they have been experiencing these issues. I discovered that they must use a particular link to get into the website. These technological issues have slowed the program, particularly yesterday when I was away."<br><br>"This isn't working. The cartoon needs to be in video format. It took far too long to load each cartoon slide. The students became disengaged and they are annoyed about the format."<br><br>"Myself and my colleague took turns reading through the cartoons which the students found very engaging"<br><br>"This is a really important topic within my class context - we have a lot of neurodiverse	"Some students were engaged, but many were not interested in the program. I modified the lesson plan to help them get more involved, but the modification helped only some of them to get more focused on the learning."<br><br>"I found this lesson quite rushed. The class interacted well with the first activity which took time away from the second activity. Both activities would be great tools for students to interact with in their own lives so would have liked more time."<br><br>"They found Activity 2 confusing. I didn't feel it was clear to them how to complete the activity. They are becoming more disengaged every week, unfortunately. It's extremely difficult to motivate them. The cartoon needs to be a cartoon with voices to actually work or in print form as a comic. There are four students who still can't gain access…the website keeps saying that their email addresses are not recognised."	"Many students were not engaged in the classroom activities. We are trying hard to engage them and make things interesting, but some are not responding. It would have been great if the contents were presented varied each week to create more student engagement and interest."<br><br>"The content is valuable but the mode of delivery via the cartoon is repetitive for them and they are now very disengaged. Even with the mP4 it takes a long time to read through it with them and stop at various points for discussion. More of an activity oriented approach with this Year 8 class would be more beneficial I feel."<br><br>"This lesson was taken by a cover teacher. I got some feedback from them that all content was covered in the time but that (as always!) there was a struggle to have enough devices for all students, so some needed to share."	"As a class, the students completed the online cartoon and then read the Students' summary. However, only some completed all the questions in the 'structured Problem-solving' activity."<br><br>"The visuals need to be in an animation form. Students tried but they found the repetitive nature of the cartoon wordy. They were also distracted by the gender identity aspects of the learning experiences and often missed the excellent points conveyed."<br><br>"This lesson was taken by a cover teacher. The feedback was that the students were slow on the start-up and struggled to get enough devices, some shared. They didn't complete all the tasks (I believe they were referring to the home tasks too) as a class but they did do some role-playing activities off-script. The students said the lesson was good and they found the content somewhat useful."	"We were very low on time towards the end (due to teacher absence in the last two weeks of term, and issues with our small number of devices). This last lesson was very rushed as we needed to get surveys done too, which was not ideal. Students were receptive to the idea of accessing material during the break if they felt they needed to."

	"Very long cartoon aids - Video's would have worked better for the students. This was drawn out a lot and was slow to click to the next slide. Learner engagement."<br><br>"They loved the cartoon style delivery, it worked really well for neurodiverse learners as at one time there is limited text on screen. The class activities were rushed due to the extra time taken to set-up which meant we had less time than planned. Overall I was very pleased with the lesson and felt the content was great, prompting important and valuable discussions."	students who exhibit black and white thinking which is often applied to their own sense of self. We had a good discussion as a follow up (me with 4-5 students) about negative thoughts and how you can recognise, accept them as thoughts only, and move on to more realistic thinking. I gave them some examples and they showed good engagement."	"This was a great activity for my class as so many of them struggle with personal organisation, burnout, etc. They often come to me concerned about their schedule and this was a great activity to launch conversations about time management and balancing social, physical and achievement tasks."			
